# Induction of Autophagy by Extract from *Corydalis heterocarpa* for Skin Anti-Aging

**DOI:** 10.3390/md22030127

**Published:** 2024-03-08

**Authors:** Kyeong Eun Yang, Soo-Bin Nam, Ga-Eun Lee, Gabsik Yang, Mee-Hyun Lee, Geul Bang, Jung Hoon Choi, Yong-Yeon Cho, Cheol-Jung Lee

**Affiliations:** 1 Biopharmaceutical Research Center, Ochang Institute of Biological and Environmental Science, Korea Basic Science Institute (KBSI), Cheongju 28119, Republic of Korea; 2 College of Pharmacy, The Catholic University of Korea, Bucheon 14662, Republic of Korea; 3 Department of Korean Medicine, College of Korean Medicine, Woosuk University, Jeonju 54986, Republic of Korea; 4 College of Korean Medicine, Dongshin University, Naju 58245, Republic of Korea; 5 Digital Omics Research Center, Ochang Institute of Biological and Environmental Science, Korea Basic Science Institute (KBSI), Cheongju 28119, Republic of Korea

**Keywords:** *Corydalis heterocarpa*, cellular senescence, anti-aging, autophagy, LRSAM1

## Abstract

The extracts of *Corydalis heterocarpa*, a salt-tolerant plant, exhibit diverse physiological properties, including anti-inflammatory, anticancer, and antiadipogenic effects. However, the anti-aging effects of *C. heterocarpa extract* (CHE) on human skin cells have not yet been investigated. In the present study, we determined that CHE inhibited senescence-associated β-galactosidase (SA-β-gal)-stained senescent human dermal fibroblasts (HDFs). Furthermore, CHE markedly suppressed the expression of major regulatory proteins involved in senescence, including p53, p21, and caveolin-1. Interestingly, CHE promoted autophagic flux, as confirmed by the formation of microtubule-associated protein 1 light chain 3B (LC3B) puncta and lysosomal activity. Notably, using RNA sequencing (RNA-seq), we showed that CHE selectively regulated the gene expression of leucine-rich repeat and sterile alpha motif-containing 1 (*LRSAM1*), an important regulator of autophagy. The adenosine-monophosphate activated protein kinase/mammalian target of rapamycin (AMPK/mTOR) pathway, which is essential for autophagy regulation, was also modulated by CHE. LRSAM1 depletion not only inhibited LC3B expression but also decreased the autophagy flux induced by CHE. Moreover, the knockdown of LRSAM1 suppressed the reversal of CHE-induced senescence in old HDFs. Collectively, our study has revealed the rejuvenating effects and molecular mechanisms of CHE, suggesting that CHE may be a promising anti-aging agent.

## 1. Introduction

The number of elderly individuals is continuously and gradually expanding. According to the World Health Organization (WHO), projected statistics indicate that by 2050, the worldwide population of individuals aged 60 years and older is expected to double, reaching a total of 2.1 billion. Additionally, it is anticipated that the population aged 80 years or older will triple from 2020 to 2050, reaching an estimated 426 million [1]. The prevalence of chronic diseases, including cranial nerve disorders, cardiovascular disease, cancer, and diabetes, is significantly associated with aging [2,3].

Cellular senescence is a significant hallmark of the aging process and is characterized by a permanent halt in cell division and irreversible cell cycle arrest [4]. Cellular senescence was first reported in 1961 by Hayflick and Moorhead, who observed senescence in human fibroblasts after repeated subculturing [5]. Senescent cells are in a state of irreversible cessation of the cell cycle while retaining their viability. Control of cell cycle arrest during cellular senescence primarily relies on regulatory pathways involving p53/p21 and p16/RB [6]. Various factors associated with senescence, including telomere attrition, reactive oxygen species (ROS), and oncogenic stress, stimulate the activation of p53 and upregulate p21 or p16, leading to the development of a senescence-associated secretory phenotype (SASP) [7,8,9]. Caveolin-1, a key structural component of caveolae, plays a critical role in regulating replicative senescence. The expression of caveolin-1 is upregulated in senescent HDFs [10], and depletion of caveolin-1 in senescent human diploid fibroblasts leads to a transformation in their morphology, resembling a non-senescent shape [11]. Furthermore, ectopic expression of caveolin-1 in bone marrow mesenchymal ST2 cells promotes the expression of p53 and p21 [12]. On the other hand, recent studies have demonstrated that senescent cells exhibit elevated autophagy [13], and altered autophagy patterns have been observed in senescent stem cells [14], suggesting the potential involvement of autophagy in cellular senescence.

Recently, compromised autophagy has been identified as a hallmark of aging [4]. Autophagy is an essential cellular mechanism responsible for maintaining cellular homeostasis and facilitating differentiation, development, and survival by selectively eliminating molecules and subcellular components such as nucleic acids, proteins, lipids, and organelles through lysosome-mediated degradation [15]. Accumulating evidence has reported that autophagy is intimately involved in the regulation of aging and lifespan. For example, transcriptomic profiling in Saccharomyces cerevisiae demonstrated that short-lived mutants exhibit impaired autophagy compared to long-lived mutants [16]. Furthermore, knockdown and/or mutation of autophagy-associated genes such as autophagy-related protein 8 (*ATG8*), autophagy-related protein 1 (*ATG1*), Beclin 1 (*Becn1*), and autophagy-related protein 7 (*ATG7*) in Caenorhabditis elegans or mice shorten lifespan [17,18]. In addition, transgenic mice overexpressing *Atg5* exhibit characteristics associated with anti-aging effects, including improved insulin sensitivity and a leaner phenotype, which are attributed to enhanced autophagy activation [19]. Therefore, it is crucial to identify agents and regulators that modulate autophagy during the aging process, specifically in relation to senescence.

Halophytes are plant species that can tolerate high salinity levels in their growth environment. Extracts or active substances derived from these plants are considered potentially useful natural products. *C. heterocarpa* is a biennial herb and salt-tolerant plant found on the sandy shores of South Korea [20]. Accumulating evidence has demonstrated that *C. heterocarpa* plays key roles in various biological activities, including anti-tumor effects [21], anti-inflammatory properties [22], anti-adipogenic effects [23], and UVB-protective characteristics [24]. However, the effect of *C. heterocarpa* extract (CHE) on aging and rejuvenation regulated by autophagy has not been studied.

In the present study, we aimed to investigate the rejuvenating effects and precise mechanism of action of CHE. The rejuvenating effects of CHE were assessed using the senescence-associated-β-galactosidase (SA-β-gal) assay, and the expression of senescence marker proteins, including p53, p21, and caveolin-1 were analyzed in senescent human dermal fibroblasts (HDFs). Furthermore, we examined the regulatory effect of CHE on autophagy using the LC3 puncta assay, lysotracker, and gene silencing. To identify the target genes of CHE, we performed RNA sequencing (RNA-seq) and analyzed the bioinformatics data. In addition, we analyzed the chemical constituents of CHE using a high-resolution liquid chromatograph-mass spectrometer (HR LC-MS).

## 2. Results

### 2.1. CHE Reverses the Cellular Senescence in Senescent HDFs

**Figure 1:**
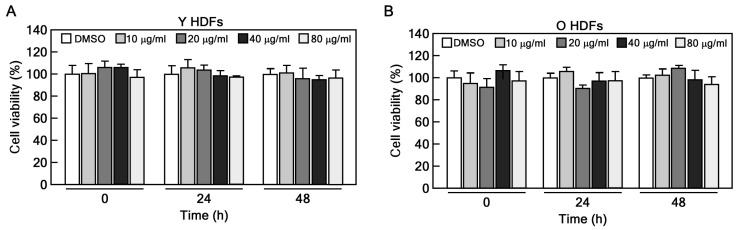
Effect of CHE on cytotoxicity. (**A**,**B**) Cell viability was assessed in young (Y HDFs) and old HDFs (O HDFs) following treatment with the indicated concentrations of CHE using a CCK-8 assay.

**Figure 2:**
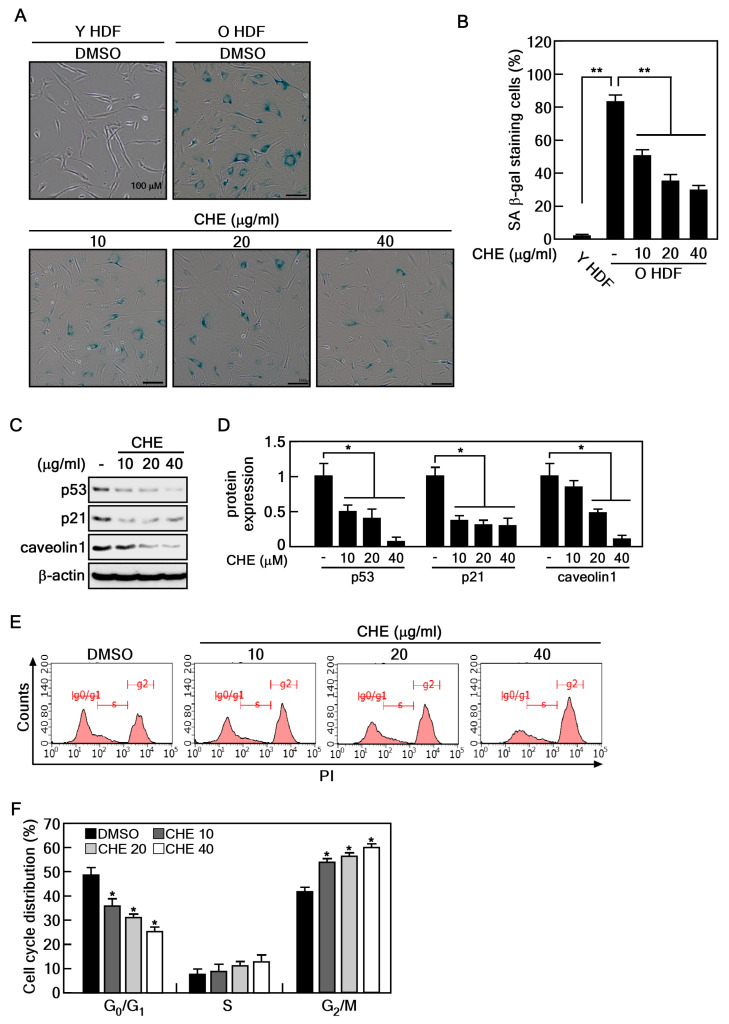
CHE reverses cellular senescence of old HDFs. (**A**,**B**) Old HDFs were treated with 10, 20, and 40 μg/mL of CHE for 48 h and subjected to a SA-β-gal assay. The stained cells were observed using microscopy and the results were normalized to the total cell number. Scale bar: 100 μm, ** *p* < 0.01. (**C**,**D**) The expression of p53, p21 and caveolin-1 in old HDFs were assessed by immunoblot assay following CHE treatment. Protein expression levels were normalized to β-actin, * *p* < 0.05. (**E**,**F**) The cell cycle distribution of old HDFs treated with CHE was evaluated using a flow cytometry assay. * *p* < 0.05.

To determine the appropriate concentration of CHE for treating HDFs, we performed a cell viability assay using young (passage number < 10) and old (passage number > 35) HDFs. The cells were treated with 10, 20, 40, and 80 μg/mL of CHE for 24 and 48 h. We determined that CHE exhibited no cytotoxicity at concentrations up to 80 ug/mL in both young and old HDFs (Figure 1A,B). Next, we conducted the SA-β-gal assay to confirm the replicative senescence in old HDFs and to investigate the potential inhibitory effects of CHE on this senescence. Approximately 80% of old HDFs were stained, whereas less than 3% of young HDFs were stained (Figure 2A,B). Importantly, CHE markedly inhibited the number of stained old HDFs compared to that of vehicle-treated old HDFs (Figure 2A,B). In senescent cells, cell cycle arrest is mediated by caveolin-1 through the p53/p21-dependent pathway [25]. In line with the report, we performed an immunoblot analysis to assess the expression of p53, p21, and caveolin-1 in CHE-treated old HDFs. These protein levels were significantly decreased after treatment with CHE (Figure 2C,D). Next, we investigated whether CHE affects the alteration of cell cycle distribution using a flow cytometry assay. The percentage of the G1 phase population was decreased, while the G2/M phase was increased in response to treatment with CHE (Figure 2E,F), indicating that CHE promotes cell cycle progression. These results suggest that CHE treatment has the potential to reverse cellular senescence by restoring normal cell cycle progression.

### 2.2. Regulation of Autophagy by CHE in Senescent HDFs

**Figure 3:**
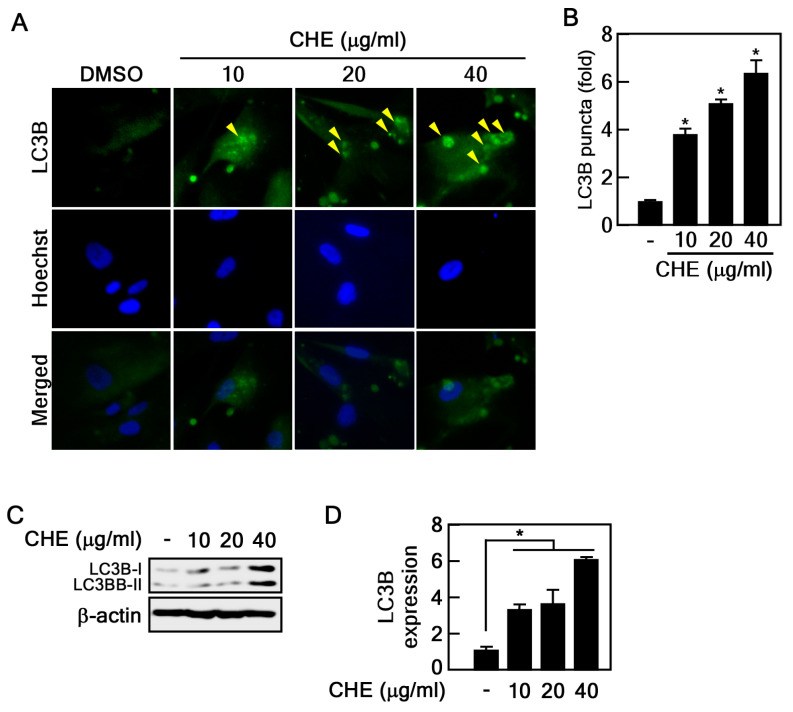
Effect of CHE on regulation of autophagy. (**A**,**B**) The induction of endogenous LC3B puncta formation (yellow triangles) by CHE was measured by ICF assay, * *p* < 0.05. (**C**,**D**) The expression of LC3B in old cells was evaluated by immunoblot assay following CHE treatment. LC3B expression level was normalized to β-actin, * *p* < 0.05.

Autophagy is generally considered to inhibit cellular senescence by eliminating damaged macromolecules and organelles. In addition, our recent results demonstrated that the activation of autophagy by Rb2, a ginsenoside, suppressed cellular senescence [10]. As CHE inhibits senescence in old HDFs (Figure 2), we hypothesized that CHE may affect the regulation of autophagy. LC3B is a well-known protein marker and an essential component for the formation of autophagosomes and autolysosomes. Its presence can be monitored using an LC3B puncta assay [26]. Therefore, we performed immunohistochemistry to detect LC3B puncta in CHE-treated HDFs. LC3B puncta were largely induced by CHE treatment compared to vehicle-treated control cells (Figure 3A,B). To further investigate the effect of CHE on LC3B, we assessed the LC3B protein expression in CHE-treated old HDFs. We found that LC3B protein levels were strongly increased by CHE treatment (Figure 3C,D). These results indicated that CHE may be a regulator of autophagy.

### 2.3. CHE Promotes Autophagic Flux

**Figure 4:**
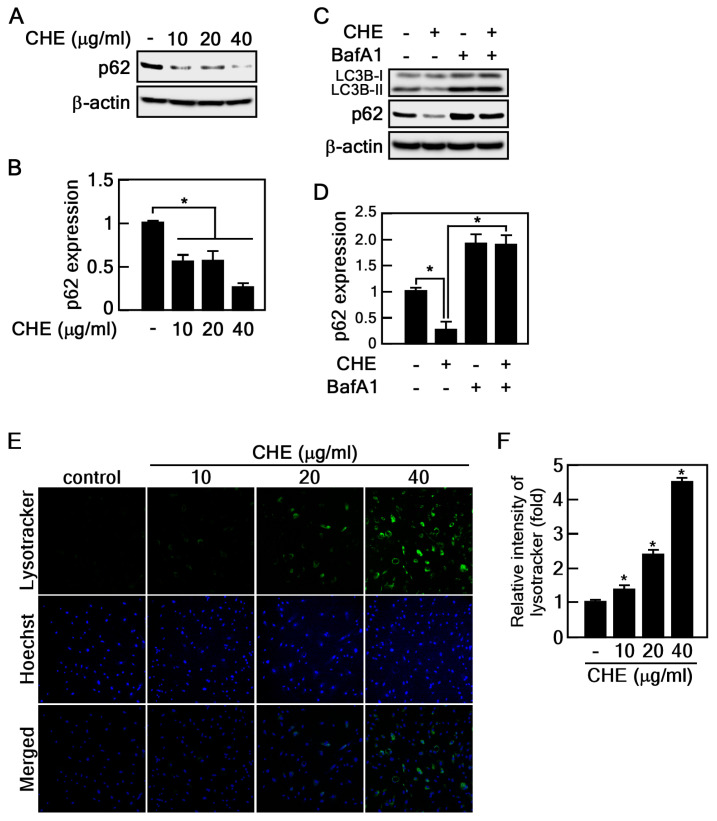
CHE regulates autophagic flux. (**A**,**B**) The expression of p62 in old HDFs was assessed by immunoblot following CHE treatment, * *p* < 0.05. (**C**,**D**) The expression of LC3B and p62 was assessed by immunoblot assay. Old HDFs were pre-treated with BafA1 (20 nM) for 2 h and then treated with CHE (20 μg/mL) for an additional 24 h, * *p* < 0.05. (**E**,**F**) Effect of CHE on lysosomal activation. Old HDFs treated with the indicated concentrations of CHE for 24 h were stained with lysotracker, and the fluorescence intensity was quantified using image J software version 1.51, * *p* < 0.05.

Elevated LC3B protein levels indicate either the formation of an autophagosome or the inhibition of autophagic flux. To assess the effect of CHE on autophagic flux, we examined changes in p62 protein expression to discern whether it induces or inhibits the process. The p62 protein serves as a mediator for the delivery of autophagic substrates to autophagosomes, and its reduction is implicated in the activation of autophagic flux [27]. Notably, CHE reduced p62 protein levels (Figure 4A,B). For further conformation, we used bafilomycin A1 (BafA1), a well-known inhibitor of the late stage of autophagic flux. The reduced protein levels of p62 and LC3B induced by CHE were obviously recovered by BafA1 treatment, indicating that CHE induces p62 degradation by autophagy (Figure 4C,D). Given that lysosomal-dependent degradation is a major mechanism in autophagy, we employed lysotracker dye, a tool widely utilized for assessing lysosomal activity associated with autophagic processes. As expected, old HDFs treated with CHE were largely stained by lysotracker, indicating that CHE enhanced lysosomal function. These results demonstrate that CHE induces autophagic flux.

### 2.4. CHE Affects Leucine-Rich Repeat and Sterile Alpha Motif-Containing 1 (LRSAM1) Expression and Adenosine-Monophosphate Activated-Protein Kinase (AMPK)-Mammalian Target of Rapamycin (mTOR) Pathway

**Figure 5:**
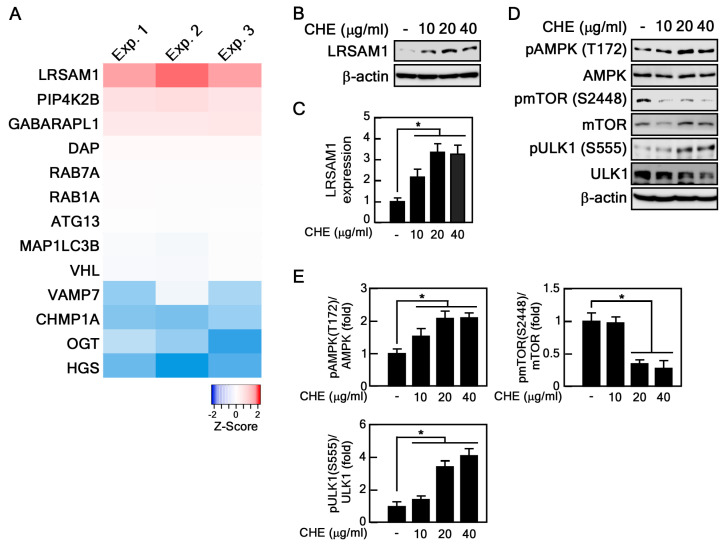
Identification of target genes and signaling pathways of CHE. (**A**) Heatmap showing RNA-seq results. The RNA was extracted from old HDFs treated with CHE (20 μg/mL) for 24 h (*n* = 3). (**B**,**C**) The expression of LRSAM1 was evaluated by immunoblot assay following CHE treatment. LRSAM1 expression level was normalized to β-actin, * *p* < 0.05. (**D**,**E**) The expression and phosphorylation level of AMPK, mTOR and ULK1 were assessed by immunoblot assay following CHE treatment. * *p* < 0.05.

Because CHE activates autophagic flux (Figure 4), we sought to identify the target genes and signaling pathway of CHE. To this end, we performed RNA sequencing on CHE-treated old HDFs. Within a pool of 24,583 human genes, we scrutinized a subset of 211 genes associated with autophagy. These genes were retrieved from QuickGO (Gene Ontology annotation, https://www.ebi.ac.uk/QuickGO/annotations (accessed on 1 January 2024)). Notably, CHE significantly increased the RNA expression of LRSAM1 (Figure 5A and Supplementary Table S1), an important regulator of autophagy [28]. Consistent with these results, CHE also increased LRSAM1 protein expression (Figure 5B,C). Given that the AMPK-mTOR pathway is a critical driver of autophagy activation [29], we investigated the effect of CHE on the regulation of this pathway. CHE inhibited phosphorylation of mTOR at Ser 2448, while it increased AMPK phosphorylation at Thr 172 (Figure 5D,E). Furthermore, the phosphorylation of ULK1 at Ser 555, which is phosphorylated by AMPK and induces its activation, was also promoted by CHE, indicating that CHE activates the AMPK-mTOR signaling pathway (Figure 5D,E). These results suggest that the activation of autophagic flux by CHE is dependent on the AMPK-mTOR pathway and LRSAM1 expression.

### 2.5. Depletion of LRSAM1 Suppresses the CHE-Induced Reversal of Cellular Senescence by Inhibiting Autophagy

**Figure 6:**
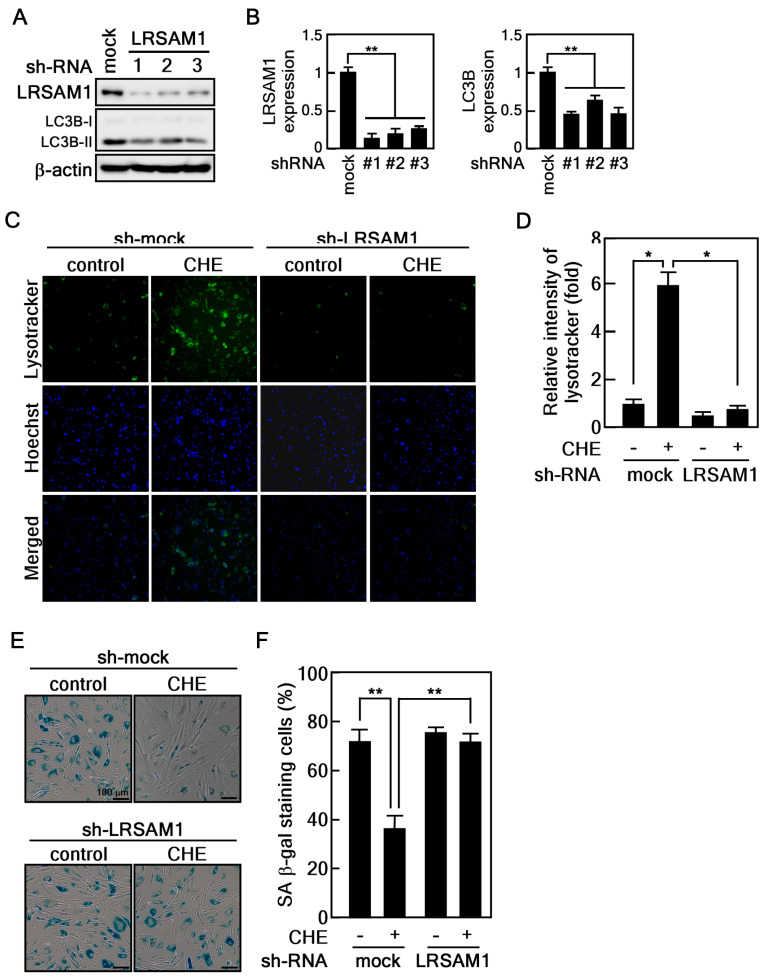
The knockdown of LRSAM1 inhibits autophagy and the CHE-induced rejuvenation in old HDFs. (**A**,**B**) Generation of old HDFs with LRSAM1 knockdown using three different of small hairpin RNAs that target LRSAM1. (**C**,**D**) Depletion of LRSAM1 suppresses lysosomal function induced by CHE. Old HDFs with LRSAM1 knockdown were treated with 20 μg/mL of CHE for 24 h. The cells were stained with lysotracker for 1 h, and the fluorescence intensity was quantified using image J software, * *p* < 0.05. (**E**,**F**) LRSAM1 is an important regulator for CHE-induced rejuvenation in old HDFs. Old HDFs with LRSAM1 knockdown were treated with 20 μg/mL of CHE for 24 h. The cells were stained with SA-β-gal solution and observed using microscopy. The results were normalized to the total cell number. Scale bar: 100 μm, ** *p* < 0.01.

To confirm the role of LRSAM1, a target gene of CHE, in regulating cellular senescence mediated by autophagy, we established LRSAM1 knockdown in old HDFs using three different small hairpin RNAs (shRNAs) targeting LRSAM1. We found that LRSAM1 depletion inhibited LC3B expression, indicating that LRSAM1 could affect autophagy regulation (Figure 6A,B). Therefore, we hypothesized that LRSAM1 is associated with the regulation of CHE-induced autophagy flux. To evaluate this hypothesis, we performed a lysotracker assay on old HDFs with LRSAM1 knockdown. Importantly, depletion of LRSAM1 abrogated CHE-induced lysosomal activity (Figure 6C,D). To further confirm the correlation between the rejuvenating effect induced by CHE and LRSAM1, we conducted a SA-β-gal assay on LRSAM1-knockdown old HDFs treated with CHE. Notably, we found that LRSAM1 depletion significantly inhibited the CHE-induced reversal of senescence in these cells (Figure 6E,F). Collectively, these data suggest that LRSAM1 plays a crucial role in the CHE-induced rejuvenating effect of senescent HDFs.

## 3. Discussion

In this study, we revealed the rejuvenating effect of CHE by conducting SA-β-gal assays, immunoblot assays, and RNA-Seq. We demonstrated that CHE reverses cellular senescence through the activation of autophagy. Mechanistically, CHE selectively increased the expression of LRSAM1 and modulated the mTOR-AMPK pathway.

Previous studies have reported that libanoridin, an ingredient of *Corydalis heterocarpa*, protected ultraviolet-B (UVB) stress through inhibition of the mitogen-activated protein kinase (MAPK) pathway and AP-1 in human keratinocyte cells [30]. In addition, (2′S)-columbianetin isolated from CHE reduced UVB-induced cell death by scavenging reactive oxygen species (ROS) generation in HaCaT keratinocytes [24]. These results allow us to consider the anti-aging effect of CHE on human skin positively, since UVB irradiation and ROS generation are major causes of skin aging [31,32]. Our data also demonstrated that CHE reverses cellular senescence and inhibits the expression of aging marker proteins such as p53, p21, and caveolin1 in human skin cells.

In addition, we identified the chemical constituents of CHE using a high-resolution liquid chromatograph-mass spectrometer (HR LC-MS) (Supplementary Figure S1 and Supplementary Table S2). Although our ethanol extract did not yield previously identified compounds such as libanoridin and (2′S)-columbianetin, which are typically extracted by methanol, we identified several compounds in CHE with potential anti-aging effects. Rutin, also known as quercetin-3-O-rutinoside, is a flavonoid glycoside found in various plants [33] and has demonstrated the ability to inhibit H_2_O_2_-induced cellular senescence and ROS generation while promoting mRNA expression of collagen in HDFs [34]. Tectoridin, a type of isoflavone glycoside isolated from the flowers of Pueraria lobata (Puerariae Flos), demonstrates antioxidative properties in vitro. This effect is achieved through the scavenging of hydroxyl and superoxide anion radicals [35]. Furthermore, tectoridin reverses lipid peroxidation induced by PM2.5 by activating the nuclear factor erythroid 2-related factor 2 (Nrf2) signaling pathway [36]. The antioxidant activity of esculin, a coumarin glucoside found in Cortex Fraxini, has been widely studied. Esculin not only inhibits the overproduction of dopamine-induced ROS in human neuroblastoma cells but also promotes the activity of superoxide dismutase (SOD) and glutathione (GSH) [37]. In addition, esculin increases the expression of Nrf2 and heme oxygenease-1 (HO-1), thereby protecting against lipopolysaccharide/D-galactosamine-induced acute liver injury in mice [38]. The decline of peripheral nerve regeneration after injury is associated with aging [39]. Isoquercitrin, also known as quercetin-3-β-D-glucoside, is a flavonoid compound prevalent in a range of medicinal and dietary plants. It has been shown to facilitate the regeneration of peripheral nerves by mitigating oxidative stress in mice with sciatic nerve crush injuries [40]. Furthermore, isoquercitrin is observed to induce autophagy in hepatocellular carcinoma cells through the activation of the AMPK/mTOR/p70S6K signaling pathway [41]. Based on these reports, CHE may potentially be used as an anti-aging agent.

Cellular protein quality control plays a crucial role in governing optimal cellular physiology through three distinct systems, such as the ubiquitin-proteasome, chaperones and autophagy [42]. Accumulating evidence has revealed that autophagy eliminates harmful components such as misfolded proteins and damaged organelles, thereby preventing aging and aging-related diseases including diabetes, metabolic diseases, and neurodegenerative diseases [43]. Our screening results demonstrated that CHE selectively promoted LRSAM1 expression among autophagy-involved genes. It has been reported that the E3 ubiquitin ligase LRSAM1 regulates ubiquitin-dependent autophagy responsible for bacterial infection [44]. Moreover, resveratrol, which is a phytochemical and well-known anti-aging compound, removed misfolded proteins associated with neurodegeneration by increasing LRSAM1 protein stability [45]. Importantly, PHD finger protein 23 (PHF23)-induced LRSAM1 degradation abrogates the autophagic process [28]. Consistent with these reports, our results demonstrate that depletion of LRSAM1 not only reduces LC3B expression but also abrogates CHE-induced autophagy flux. Furthermore, old HDFs with LRSAM1 knockdown did not show a decrease in staining in the SA-β-gal assay, even after CHE treatment, indicating that LRSAM1 is involved in regulating the reversal of cellular senescence induced by CHE. Thus, our study provides evidence that LRSAM1 could play an essential role in rejuvenation by regulating autophagy.

The AMPK/mTOR/Ulk1 signaling pathway is essential for energy-sensing and autophagy regulation [46]. The activation of AMPK directly phosphorylates Ulk1, resulting in the promotion of autophagy [29]. The Ulk1 complex, consisting of Ulk1, autophagy-related protein 13 (ATG13), focal adhesion kinase family interacting protein of 200 kDa (FIP200), and ATG101, is a major initiator for the formation of an autophagosome [47]. mTORC1 is a pivotal regulator in the process of autophagy through phosphorylation of Ulk1, which leads to the inactivation of the Ulk1 complex [29]. Furthermore, death-associated protein 1 (DAP1), a negative regulator of autophagy, is phosphorylated and activated by mTORC1 [48]. Therefore, mTORC1 has an inhibitory role in autophagy. Here, we show that CHE inhibits mTORC1, while it induces the AMPK/Ulk1 pathway, suggesting that CHE could be an activator of autophagy. Thus, further studies are needed to gain a comprehensive understanding of the precise mechanism of CHE’s effect on autophagy, particularly regarding the connection between LRSAM1 and the AMPK/mTOR/Ulk1 pathway.

**Figure 7:**
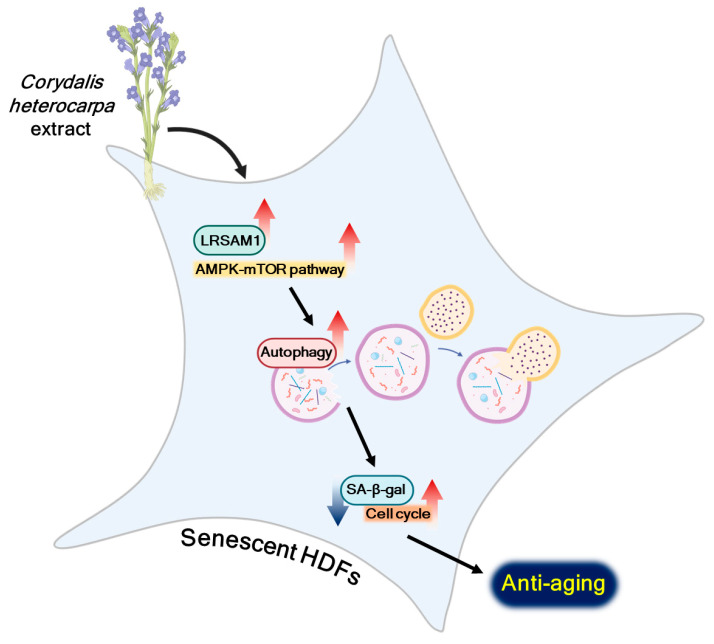
Schematic summary of the proposed mechanism.

In conclusion, our study has revealed the rejuvenating properties of CHE and elucidated the underlying molecular mechanism by which it induces autophagy, specifically through the regulation of LRSAM1 expression and the AMPK-mTOR pathway (Figure 7). These findings suggest the potential of CHE as a promising candidate for an anti-aging agent.

## 4. Materials and Methods

### 4.1. Corydalis Heterocarpa Extract

CHE (MABIK NP60190015) was provided by the National Marine Biodiversity Institute of Korea (MABIK). The *C. heterocarpa* was collected from the coastline of Yeosu, Jeollanam-do, Korea, in June 2017. To obtain the extract, the *C. heterocarpa* specimens were first subjected to three rounds of washing with tap water and then freeze-dried using a freeze dryer (OPERON FDT-8650). The freeze-dried biological sample powder (30 g) was pulverized and suspended in 400 mL of 70% ethanol (EtOH), then subjected to three successive extractions at room temperature using an ultrasonic extractor (DAIHAN WUC-N30H) operating at a frequency of 40 kHz for a duration of 60 min per extraction cycle. The resulting mixture was then filtered using filter paper (Whatman 2V Folded Filters Diameter 320 mm 100 Circles, Product 1202-320), and the extract was obtained through evaporation using a rotary evaporator (Buchi CH/R-210).

### 4.2. Cell Culture and Treatment

Primary human dermal fibroblasts (HDFs), obtained from the Coriell Institute for Medical Research (cell line AG08498), were cultured in Dulbecco’s Modified Eagle Medium (DMEM) supplemented with 10% fetal bovine serum (FBS) and 1% antibiotic-antimycotic solution. Subculturing of HDFs was performed at a 1:4 ratio when the cells reached approximately 80–90% confluence in 100 mm cell culture dishes, and they were maintained until reaching senescence. Cultures of young cells corresponded to passages 8–10, while those of old cells corresponded to passages 34–36. For treatment with CHE, the extract was dissolved in dimethyl sulfoxide (DMSO) and applied at the indicated dose and for the specified incubation time.

### 4.3. CCK-8 Assay

HDFs were seeded at 3 × 10^3^ cells/well in 96-well plates and incubated for 24 h. The cells were treated with 10, 20, 40, and 80 μg/mL of CHE for 24 h and 48 h. Subsequently, 10 μL of CCK-8 solution was added to each well and incubated for 1 h at 37 °C. After gentle shaking, the absorbance was measured at 450 nm using a microplate reader (Infinite 200 PRO, Tecan, Männedorf, Switzerland).

### 4.4. Senescence-Associated β-galactosidase Staining Assay

The young and old HDFs were seeded at 3 × 10^4^/well into 12-well plates and cultured. The cells were exposed to the indicated concentrations of CHE (10, 20, and 40 μg/mL). After incubation for 48 h, SA-β-gal staining was performed using a SA-β-gal staining kit according to the manufacturer’s instructions. In brief, the fixed cells were stained with 500 μL of β-galactosidase staining solution for 16 h at 37 °C. Stained cells were imaged at a magnification of ×100 using a microscope equipped with a camera (Eclipse Ti2-U, Nikon, Tokyo, Japan). The quantification of SA-β-gal-positive cells was performed by counting cells in three randomly selected fields.

### 4.5. Western Blotting

Old HDFs were treated with CHE at the indicated concentrations, and the cells were disrupted by EBC buffer (120 mM NaCl, 0.5% NP-40, 50 mM Tris-Cl, pH 8.0) containing a protease inhibitor cocktail. The whole protein lysate was obtained by centrifugation at 13,000× *g* for 10 min. Protein concentration was determined using the BCA protein kit (Thermo Fisher Scientific, Vantaa, Fin-land). Equal amounts of protein (30 μg) were separated on 8–15% SDS-PAGE gels and transferred to a nitrocellulose membrane. The membrane was blocked using 5% skim milk in tris-buffer saline with 0.1% Tween-20 (TBST) for 1 h at RT and then incubated with the indicated primary in 3% skim milk overnight at 4 °C. After washing three times with TBST, the blots were hybridized with secondary antibodies for 1 h at RT. Protein expression bands were visualized using Azure Biosystems and quantified using the image J program. The antibodies used in this study are listed in the table below (Table 1).

### 4.6. LC3B Puncta Formation

Old HDFs were seeded into a chamber slide, cultured, and treated with the indicated concentrations of CHE for 24 h. The cells were fixed with 4% formalin and permeabilized using 0.5% Triton X-100/PBS, followed by blocking using 3% BSA/Tween-20/PBS for 1 h at RT. Subsequently, the cells were subjected to overnight hybridization with a primary antibody against LC3B in 3% BSA/PBS at 4 °C. After washing three times, the cells were incubated with an Alexa 488-conjugated secondary antibody for 1 h at RT. The visualization of LC3B puncta was accomplished using a fluorescence microscope. The intensity was measured using the image J program.

### 4.7. Measuring Autophagy-Associated Lysosomal Activity

Old HDFs were seeded into chamber slide and treated with 10, 20, and 40 μg/mL of CHE for 24 h. The cells were then treated with LysoTracker Green DND-26 (100 nM) for 1 h, and the fluorescence was observed using a fluorescence microscope. The intensity of Lysotracker and Hoechst was measured using the image J program, and the lysosomal activity was normalized to Hoechst intensity.

### 4.8. Identification of Compounds in CHE Using LC/MS

The CHE was analyzed using a Thermo Orbitrap 120 mass spectrometer (Thermo Fisher Scientific; Waltham, MA, USA) coupled to the AQUITY UPLC system. The chromatographic separation was performed on a Hypersil GOLD C18 column (150 mm × 2.1 mm, 3 µm, Thermo Fisher). The mobile phases consisted of 0.1% formic acid in ultrapure water (A) and 0.1% formic acid in acetonitrile (B). The flow rate and injection volume were 0.2 mL/min and 3 μL, respectively. The total chromatographic separation runtime was 20 min. The mass spectra were obtained in negative ion mode using an ESI source. The MS conditions were optimized as follows: the ion transfer tube temperature was set to 320 °C and the vaporizer temperature to 275 °C, with an acquisition mass range for *m*/*z* of 100–1500 in negative ionization mode. Compounds from CHE were rapidly identified based on their precise molecular masses and MS^2^ fragment ions using Compound Discoverer software version 3.3 (CD, Thermo Fisher Scientific, Chicago, IL, USA). The ions [2M-H]^−1^, [M-2H]^−2^ and [M-H]^−1^ were set as the base peaks in CD, and the minimum peak intensity threshold was set to 60,000 to collect the MS data. The minimum number of isotopic peaks was 1, the minimum scan point was 5, and the MS tolerance was 5 ppm. The analysis of compounds from CHE identification were performed through the use of spectral libraries and compound databases available on the mzCloud database (https://www.mzcloud.org (accessed on 1 January 2024)). This experiment and analysis were performed at the Korea Polymer Testing & Research Institute (Koptri, Seoul, Republic of Korea).

### 4.9. Cell Cycle Distribution Analysis

Old HDFs were seeded at 5 × 10^5^ cells in 100 mm dishes and cultured overnight. The cells were treated with the indicated concentrations of CHE for 24 h, then trypsinized and fixed with 70% ethanol at −20 °C for at least 2 h. Subsequently, the cells were washed with cold PBS and treated with an RNase/propidium iodide solution for 15 min at RT in dark conditions. The cell cycle distribution was analyzed using the Guava easyCyte flow cytometer (Merck Millipore, Burlington, MA, USA).

### 4.10. RNA-Sequencing

Total RNA was extracted using Trizol reagent (Invitrogen) according to the manufacturer’s protocol. RNA quality was evaluated by Agilent 2100 bioanalyzer using the RNA 6000 Nano Chip (Agilent Technologies, Amstelveen, The Netherlands). The quantification of RNA was measured by an ND-2000 spectrophotometer (Thermo Fisher Scientific; Waltham, MA, USA). For control and test RNAs, the construction of the library was performed using the QuantSeq 3′ mRNA-Seq Library Prep Kit (Lexogen, Inc., Vienna, Austria) according to the manufacturer’s instructions. In brief, 500 ng of total RNA were prepared, an oligo-dT primer containing an Illumina-compatible sequence at its 5′ end was hybridized to the RNA and reverse transcription was performed. After degradation of the RNA template, second-strand synthesis was initiated by a random primer containing an Illumina-compatible linker sequence at its 5′ end. The double-stranded library was purified by using magnetic beads to remove all reaction components. The library was amplified to add the complete adapter sequences required for cluster generation. The finished library is purified from PCR components. High-throughput sequencing was performed as single-end 75 sequencing using NextSeq 500 (Illumina, Inc., San Diego, CA, USA). For data analysis, QuantSeq 3′ mRNA Seq reads were aligned using Bowtie2 (Langmead and Salzberg, 2012). Bowtie2 indices were either generated from the genome assembly sequence or the representative transcript sequences for alignment to the genome and transcriptome. The alignment file was used for assembling transcripts, estimating their abundances and detecting differential expression of genes. Differentially expressed genes were determined based on counts from unique and multiple alignments using coverage in Bedtools (Quinlan AR, 2010). The RC (Read Count) data were processed based on the Quantile normalization method using EdgeR within R (R development Core Team, 2020) using Bioconductor (Gentleman et al., 2004). Gene classification was based on searches conducted by DAVID (http://david.abcc.ncifcrf.gov/ (accessed on 1 January 2024)) and Medline databases (http://www.ncbi.nlm.nih.gov/ (accessed on 1 January 2024)). Data mining and graphic visualization were performed using ExDEGA (Ebiogen Inc., Seoul, Republic of Korea).

### 4.11. Materials

Protease inhibitor cocktail (P8340), phosphatase inhibitor cocktail 2 (P5726), phosphatase inhibitor cocktail 3 (P0044), bovine serum albumin (A7906), formaldehyde solution (F8775), and Triton X-100 (X1100) were purchased from Sigma-Aldrich (Burlington, Massachusetts, USA). Fetal bovine serum (SH30919.03) and antibiotics-antimycotic (SV30079.01) were purchased from HyClone. LysoTracker Green DND-26 (L7526), Hoechst 33342 (H3570) and BCA protein assay kit (23225) were purchased from Thermo Fisher Scientific. The SA-β-gal staining kit (9860) was purchased from Cell Signaling Technology. Dulbecco’s Modified Eagle’s Medium (LM001-05) was purchased from WELGENE.

### 4.12. Statistical Analysis

We conducted a minimum of three independent repetitions for all experiments. Statistical analyses were carried out using the Student’s *t*-test with Graph-Pad Prism software version 10.2.1 (GraphPad, La Jolla, CA, USA). Results are expressed as means ± standard deviation. Statistical significance was defined as *p*-values less than 0.05 or 0.01.

## Figures and Tables

**Table 1:** The list of antibodies used in this study.

Antibodies	Company	Catalog
p53	Santa Cruz Biotechnology	sc-126
p21	Cell Signaling Technology	2946
caveolin-1	Cell Signaling Technology	3238
β-actin	Santa Cruz Biotechnology	sc-47778
LC3B	Novus Biologicals	NB100-2220
p62	Novus Biologicals	NBP1-48320
p-AMPK	Cell Signaling Technology	2531
AMPK	Cell Signaling Technology	2532
p-mTOR	Cell Signaling Technology	5536
mTOR	Cell Signaling Technology	2972
p-ULK1	Cell Signaling Technology	6888
ULK1	Cell Signaling Technology	4773
LRSAM1	Cell Signaling Technology	28405

## Data Availability

Data sharing will be available upon written and reasonable request from the corresponding authors.

## Supplementary Materials

The following supporting information can be downloaded at: https://www.mdpi.com/article/10.3390/md22030127/s1, Figure S1: High resolution liquid chromatograph-mass spectrometer (HR LC-MS) profile of *C. heterocarpa* extract; Table S1: List of target genes involved in *C. heterocarpa* extract-regulated autophagy. Table S2: Chemical composition of *C. heterocarpa* extract using HR LC-MS analysis methods.
