# Supplementary material for: Induction of Autophagy by Extract from Corydalis heterocarpa for Skin Anti-Aging
Source: Mar Drugs. 2024 Mar 8;22(3):127. doi: 10.3390/md22030127 (PMC10972372; doi:10.3390/md22030127)
Supplement: Supplementary file 1 [file marinedrugs-22-00127-s001.zip › marinedrugs-2867145-supplementary.pdf]

[Supplementary Information]

Induction of autophagy by extract from *Corydalis heterocarpa* for skin anti-aging

Kyeong Eun Yang[1], Soo-Bin Nam[1,2], Ga-Eun Lee[2], Gabsik Yang[3], Mee-Hyun Lee[4], Geul Bang[5], Jung Hoon Choi[5], Yong-Yeon Cho[2], Cheol-Jung Lee[1*]

[1] Biopharmaceutical research center, Ochang Institute of Biological and Environmental Science, Korea Basic Science Institute (KBSI), Cheongju 28119, Republic of Korea.

[2] College of Pharmacy, The Catholic University of Korea, Bucheon 14662, Republic of Korea

[3] Department of Korean Medicine, College of Korean Medicine, Woosuk University, Jeonju 54986, Republic of Korea.

[4] College of Korean Medicine, Dongshin University, Naju 58245, Republic of Korea.

[5] Digital omics research center, Ochang Institute of Biological and Environmental Science, Korea Basic Science Institute (KBSI), Cheongju 28119, Republic of Korea.

*Corresponding Authors: Cheol-Jung Lee, Ph. D., E-mail: veritas0613@kbsi.re.kr

Content

1. Supplementary Figure

3. Supplementary Table

Supplementary Figure

Supplementary Figure S1. High resolution liquid chromatograph-mass spectrometer (HR LC-MS) profile of *C. heterocarpa* extract.

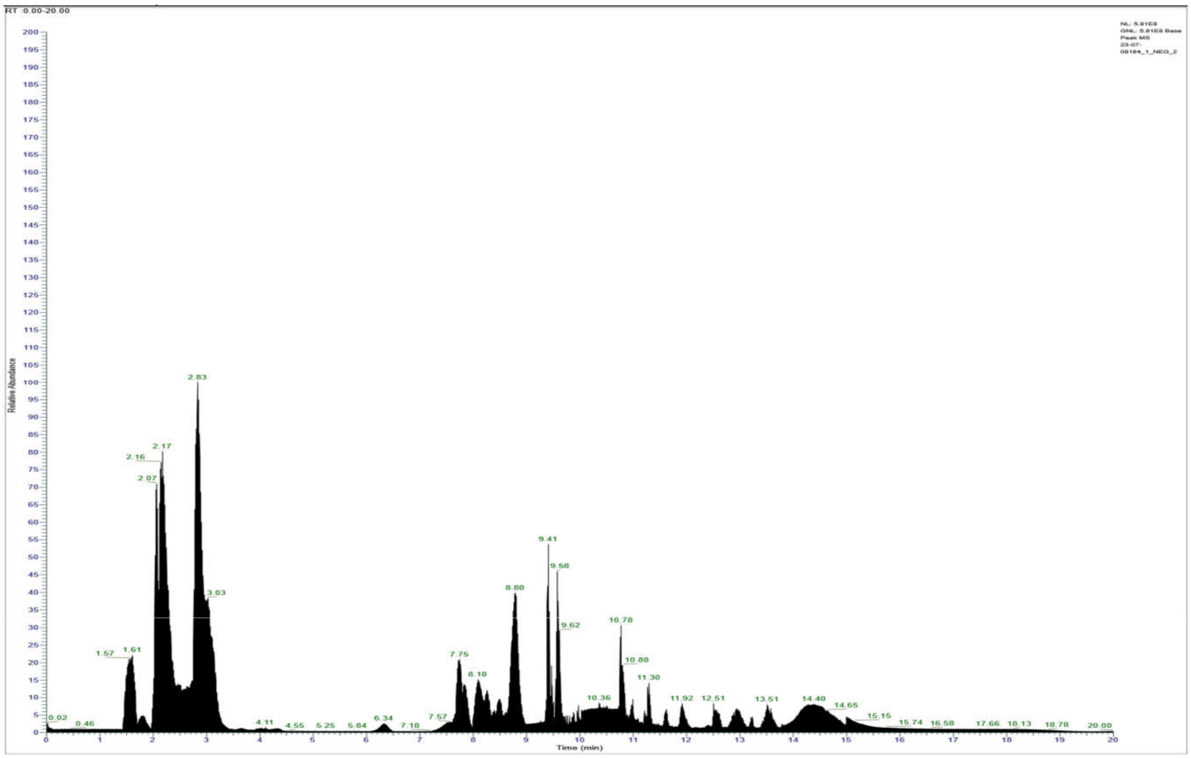

Supplementary Table

Supplementary Table S1. List of target genes involved in *C. heterocarpa* extract-regulated autophagy

	Normalized Data (log2) n=3					
	control-1	control-2	control-2	CH-1	CH-2	CH-3
LRSAM1 (Leucine Rich Repeat And Sterile Alpha Motif Containing 1)	4.335	3.992	4.219	4.893	5.309	4.783
PIP4K2B (Phosphatidylinositol-5-phosphate 4-kinase type-2 beta)	6.198	6.109	6.210	6.556	6.577	6.364
GABARAPL1 (GABA Type A Receptor Associated Protein Like 1)	7.916	8.122	7.982	8.143	8.348	8.293
DAP (Death-associated protein)	8.085	8.152	8.112	8.005	8.017	8.009
RAB7A (Ras-Related Protein Rab-7a)	8.551	8.497	8.555	8.318	8.423	8.297
RAB1A (Ras-Related Protein Rab-1a)	7.625	7.659	7.760	7.487	7.399	7.523
ATG13 (Autophagy Related 13)	5.727	5.865	5.665	5.464	5.589	5.405
MAP1LC3B (Microtubule Associated Protein 1 Light Chain 3 Beta)	7.928	7.858	7.817	7.628	7.427	7.652
VHL (Von Hippel-Lindau Tumor Suppressor)	6.104	5.999	5.873	5.777	5.638	5.672
VAMP7 (Vesicle Associated Membrane Protein 7)	3.658	3.469	3.442	2.990	3.281	2.885
CHMP1A (Charged Multivesicular Body Protein 1A)	6.603	6.588	6.484	6.001	5.912	6.047
OGT (O-Linked N-Acetylglucosamine (GlcNAc) Transferase)	4.979	5.033	5.300	4.729	4.550	4.304
HGS (Hepatocyte Growth Factor-Regulated Tyrosine Kinase Substrate)	6.842	7.189	6.984	6.417	6.224	6.381

Supplementary Table S2. Chemical composition of *C. heterocarpa* extract using HR LC-MS analysis methods

**Compounds**	**Retention Time (min)**	**Molecular Weight**
2-Furoic acid	2.17	112.015
Citric acid	2.83	192.026
Tectoridin	2.89	462.114
Filgotinib	3.03	425.152
Esculin	7.74	340.078
Methoxy-myricetin-3-O-hexosyl-7-Odeoxyhexosyl(1-2)deoxyhexoside	8.14	786.220
Rutin	8.26	610.152
Quercetin-3-β-D-glucoside	8.38	464.094
Cribrarione A	8.64	278.042
Divirensol A	9.40	578.273
Corchorifatty acid F	9.41	328.224
Perfluorobutanoic acid	9.58	213.985
Inotilone	9.62	218.057
SuncheonosideA	11.3	400.154
